# Chrysoeriol Prevents TNFα-Induced CYP19 Gene Expression via EGR-1 Downregulation in MCF7 Breast Cancer Cells

**DOI:** 10.3390/ijms21207523

**Published:** 2020-10-12

**Authors:** Dong Yeong Min, Euitaek Jung, Sung Shin Ahn, Young Han Lee, Yoongho Lim, Soon Young Shin

**Affiliations:** 1 Department of Biological Sciences, Sanghuh College of Lifesciences, Konkuk University, Seoul 05029, Korea; alsehdeodehd@naver.com (D.Y.M.); mylife4sci@naver.com (E.J.); wendy713@konkuk.ac.kr (S.S.A.); yhlee58@konkuk.ac.kr (Y.H.L.); 2 Cancer and Metabolism Institute, Konkuk University, Seoul 05029, Korea; 3 Division of Bioscience and Biotechnology, BMIC, Konkuk University, Seoul 05029, Korea; yoongho@konkuk.ac.kr

**Keywords:** breast cancer, chrysoeriol, CYP19 aromatase, EGR-1, TNFα

## Abstract

Estrogen overproduction is closely associated with the development of estrogen receptor-positive breast cancer. Aromatase, encoded by the cytochrome P450 19 (CYP19) gene, regulates estrogen biosynthesis. This study aimed to identify active flavones that inhibit CYP19 expression and to explore the underlying mechanisms. CYP19 expression was evaluated using reverse transcription PCR, quantitative real-time PCR, and immunoblot analysis. The role of transcription factor early growth response gene 1 (EGR-1) in CYP19 expression was assessed using the short-hairpin RNA (shRNA)-mediated knockdown of EGR-1 expression in estrogen receptor-positive MCF-7 breast cancer cells. We screened 39 flavonoids containing 26 flavones and 13 flavanones using the EGR1 promoter reporter activity assay and observed that chrysoeriol exerted the highest inhibitory activity on tumor necrosis factor alpha (TNFα)-induced EGR-1 expression. We further characterized and demonstrated that chrysoeriol inhibits TNFα-induced CYP19 expression through inhibition of extracellular signal-regulated kinase 1/2 (ERK1/2)-mediated EGR-1 expression. Chrysoeriol may be beneficial as a dietary supplement for the prevention of estrogen receptor-positive breast cancer, or as a chemotherapeutic adjuvant in the treatment of this condition.

## 1. Introduction

Breast cancer is the most common form of cancer diagnosed in women worldwide. Approximately 90% of all breast cancers are sensitive to female hormones, such as estrogen and progesterone, while two-thirds of postmenopausal breast cancers are estrogen-dependent [1]. Estrogen plays a pivotal role in the proliferation of breast epithelial cells. Abnormal local overproduction of estrogen in breast tissues is implicated in breast cancer pathogenesis.

Estrogen biosynthesis is regulated by aromatase, which is encoded by the cytochrome P450 19 gene (CYP19) located on chromosome 15q21.1. CYP19 aromatase, also known as estrogen synthetase or estrogen synthase, catalyzes the aromatization of androgen to estrogen in the endoplasmic reticulum [2]. There is a significant correlation between tumor incidence and aromatase activity in breast tissue [3], and aberrant aromatase expression is closely associated with breast cancer development [1], suggesting the etiological role of estrogen in breast cancer incidence. As the expression of the estrogen receptor (ER) is considerably high in breast tumors, targeting the ER or estrogen deprivation has been recognized as a potential strategy for the treatment or prevention of breast cancer. Indeed, inhibition of estrogen synthesis by aromatase inhibitors or ER antagonists prevents breast cancer development in postmenopausal women [4,5].

Several clinical studies have demonstrated that in the treatment of early to advanced ER-positive breast cancer, estrogen deprivation therapy targeting aromatase is more effective than tamoxifen (Nolvadex, AstraZeneca, Macclesfield, United Kingdom), which functions as a selective estrogen receptor modulator (SERM) [1,6,7,8]. However, over 20% of ER-positive breast cancer patients treated with aromatase inhibitors develop drug resistance [9].

While the underlying mechanisms responsible for the development of resistance to aromatase inhibitors remain elusive, several potential mechanisms have been suggested [10]. A recent study demonstrated that 21.5% of aromatase inhibitor-treated patients exhibited CYP19 amplification [11], suggesting that aromatase upregulation may be associated with drug resistance. Therefore, we hypothesized that the inhibition of aberrant aromatase expression is necessary for estrogen deprivation therapy in the treatment of ER-positive breast cancers, and that natural food ingredients that exert an inhibitory effect on aromatase expression may be beneficial for the chemoprevention of ER-positive breast cancers, or can serve as chemotherapeutic adjuvants in combination with aromatase inhibitors or ER antagonists.

Flavonoids are the most common polyphenolic compounds that are relatively abundant in the human diet, and generally exhibit multiple pharmacological effects, including antioxidant, anti-inflammation, and anti-cancer activities, such as prevention of breast cancer [12,13]. To date, over 5000 natural flavonoids have been characterized. Among them, some flavones (for example, apigenin and eriodictyol) and flavanones (for example, hesperidine and hesperetin) are known to inhibit aromatase activity [14,15,16,17,18]. However, there is limited information regarding the flavonoid compounds that inhibit CYP19 expression.

In premenopausal women, CYP19 aromatase levels are the highest in the ovarian granulosa cells, whereas in postmenopausal women, adipocytes are the primary site of aromatase expression [19]. In breast cancer cells, proinflammatory cytokines, such as tumor necrosis factor alpha (TNFα), upregulate CYP19 expression in breast cancer cells [20,21], suggesting the autocrine stimulation of estrogen in ER-positive breast cancer cells. TNFα is produced by adipocytes, tumor cells, and tumor-associated fibroblasts, and plays a critical role in the development of breast cancer, including cancer cell proliferation, invasion, metastasis, and angiogenesis [22].

In this study, we investigated the role of the transcription factor early growth response gene 1 (EGR-1) in the induction of CYP19 expression in response to TNFα stimulation in ER-positive MCF-7 breast cancer cells. Additionally, we observed that chrysoeriol (4′,5,7-trihydroxy-3′-methoxyflavone) potently reduces the accumulation of TNFα-induced aromatase through the inhibition of EGR-1-mediated CYP19 expression.

## 2. Results and Discussion

### 2.1. TNFα Induced CYP19 Expression

The tumor microenvironment plays a critical role in breast cancer development [23]. TNFα is a major inflammatory cytokine in the breast cancer microenvironment [22]. Previous studies have demonstrated that TNFα induces CYP19 aromatase expression in the breast adipose tissue [24,25].

**Figure 1:**
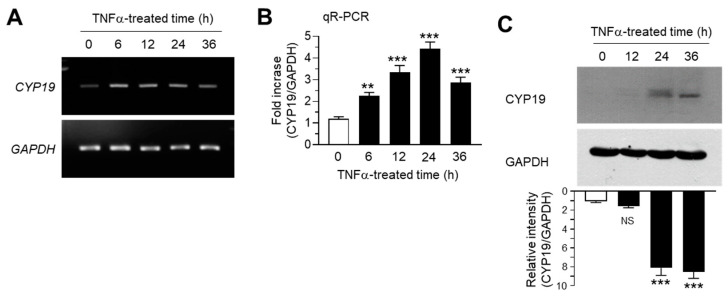
Effect of tumor necrosis factor alpha (TNFα) on cytochrome P450 19 gene (CYP19) expression. MCF-7 cells were treated with 10 ng/mL TNFα for various durations (0–36 h). (**A**,**B**) *CYP19* mRNA levels were determined using RT-PCR (**A**) and qR-PCR (**B**). Glyceraldehyde 3-phosphate dehydrogenase (GAPDH) mRNA levels were used as an internal control. (**C**) CYP19 aromatase levels were determined by immunoblot analysis. GAPDH levels were used as internal control. Band intensities were measured using the ImageJ software. Bars represent the mean ± SD (*n* = 3). NS, not significant, ** *p* < 0.01, *** *p* < 0.001 versus control (time 0) by Dunnett’s multiple comparisons test. qR-PCR, quantitative real-time PCR.

We determined the effect of TNFα on CYP19 expression in MCF-7 cells. MCF-7 cells were treated with 10 ng/mL TNFα for various time periods. Conventional RT-PCR analysis revealed that CYP19 mRNA expression increased within 6 h after TNFα treatment, followed by a gradual decrease through 36 h, whereas the mRNA levels of glyceraldehyde 3-phosphate dehydrogenase (GAPDH) remained unaltered (Figure 1A). Quantitation of mRNA levels using quantitative real-time PCR analysis (qR-PCR) revealed that compared to the basal levels, CYP19 mRNA levels were significantly (all *p* < 0.001) increased 2.27 ± 0.153-, 3.33 ± 0.321-, 4.43 ± 0.306-, and 2.87 ± 0.252-fold upon TNFα treatment for 6, 12, 24, and 36 h, respectively (Figure 1B). We further confirmed the accumulation of CYP19 aromatase by immunoblot analysis. CYP19 aromatase proteins peaked within 24 h after treatment and remained elevated for at least 36 h (Figure 1C). These results confirmed that TNFα upregulates CYP19 aromatase expression at the transcript level in MCF-7 breast cancer cells.

### 2.2. TNFα Upregulated EGR-1 Expression in MCF-7 Breast Cancer Cells

**Figure 2:**
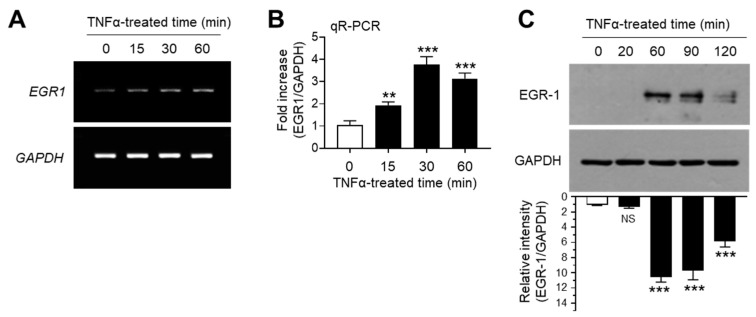
Effect of TNFα on early growth response gene 1 (EGR-1) expression. MCF-7 cells were treated with 10 ng/mL TNFα for various durations (0–60 min). (**A**,**B**) EGR1 mRNA levels were determined by RT-PCR (**A**) and qR-PCR (**B**). GAPDH mRNA levels were used as an internal control. (**C**) EGR-1 levels were measured by immunoblot analysis. GAPDH levels were used as an internal control. Band intensities were measured using the ImageJ software. Bars represent the mean ± SD (*n* = 3). ** *p* < 0.01, *** *p* < 0.001 by Dunnett’s multiple comparisons test. NS, not significant; qR-PCR, quantitative real-time PCR.

The transcription factor EGR-1 is an immediate early response protein that is rapidly induced in response to diverse extracellular signals, such as growth factors, DNA damage, and inflammatory cytokines [26,27,28,29]. Previous studies have demonstrated that EGR-1 expression is upregulated in response to TNFα stimulation in several cell types [30,31,32,33]. Consistent with previous findings, we observed the increase in EGR1 mRNA expression upon TNFα stimulation in MCF7 cells, as revealed by RT-PCR (Figure 2A) and qR-PCR (Figure 2B). Concordant with mRNA levels, immunoblotting studies yielded similar results (Figure 2C). These results demonstrated that EGR-1 expression is upregulated in response to TNFα stimulation in MCF-7 breast cancer cells.

EGR-1 plays an essential role in tumor development in several cancer cells, including breast cancer [34,35,36,37]. The mitogenic chemokine growth-regulated oncogene (GROα), also known as the C-X-C motif ligand 1 (CXCL1) or melanoma growth stimulating activity alpha (MGSA-α), elicits mitogenic properties via EGR-1 induction [36]. In addition, EGR-1 enhances GROα and matrix metalloproteinase-9 (MMP-9) expression in response to TNFα stimulation in HeLa cells [38,39]. However, the role of TNFα-induced EGR-1 expression in CYP19 expression in breast cancer cells remains unknown.

### 2.3. Silencing of EGR-1 Abrogated TNFα-Induced CYP19 Expression

**Figure 3:**
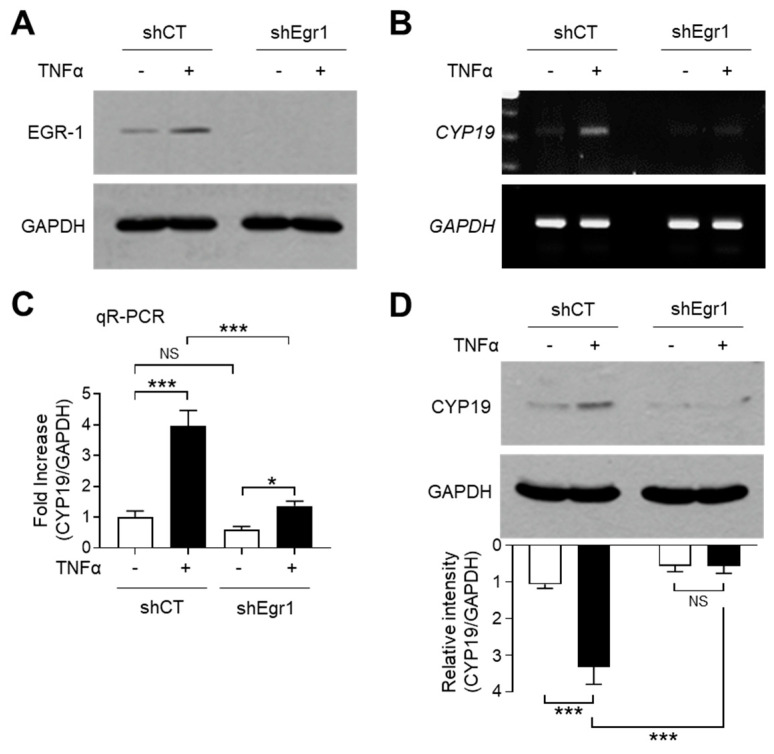
Effect of EGR-1 silencing on CYP19 expression. (**A**) EGR-1 expression in stably transfected MCF-7 cells expressing scrambled control (shCT) or EGR1 short-hairpin RNA (shEgr1) was verified by immunoblotting. Exponentially growing MCF-7 variant cells were cultured in the presence or absence of 10 ng/mL TNFα for 1 h. The cells were then harvested and analyzed by immunoblotting using anti-EGR-1 antibody. GAPDH was used as an internal control. (**B**–**D**) MCF-7 cells expressing shCT or shEgr1 were treated with 10 ng/mL TNFα for 24 h. CYP19 mRNA levels were determined by RT-PCR (**B**) and qR-PCR (**C**). *GAPDH* mRNA levels were used as an internal control. CYP19 aromatase levels were measured by immunoblot analysis (**D**). GAPDH levels were used as an internal control. Band intensities were measured using the ImageJ software. Bars represent the mean ± SD (*n* = 3). * *p* < 0.05, *** *p* < 0.001 by Dunnett’s multiple comparisons test. NS, not significant; qR-PCR, quantitative real-time PCR.

To investigate whether TNFα-induced EGR-1 expression is linked to CYP19 aromatase expression, we used MCF-7 variant cell lines expressing lentiviral short-hairpin RNA (shRNA) against EGR-1 (shEgr1) or the scrambled control (shCT). The stable knockdown of EGR-1 after TNFα stimulation was confirmed by immunoblot analysis (Figure 3A). RT-PCR analysis revealed that EGR-1 silencing abrogated TNFα-induced CYP19 mRNA expression (Figure 3B). Quantitation of mRNA using qR-PCR revealed that compared to the MCF7/shCT cells, basal CYP19 mRNA levels were significantly reduced to 0.600 ± 0.100-fold, while TNFα-induced CYP19 mRNA levels were significantly reduced from 3.97 ± 0.503-fold to 1.37 ± 0.153-fold in MCF7/shEgr1 cells (Figure 3C). Immunoblot analysis revealed that the level of CYP19 aromatase reduced significantly in MCF7/shEgr1 cells compared to that in MCF7/shCT cells (Figure 3D). These results suggest that EGR-1 plays a critical role in both basal and TNFα-induced CYP19 expression in MCF-7 cells.

### 2.4. Screening of Natural Flavone and Flavanone Compounds Inhibiting TNFα-Induced EGR-1 Expression

**Figure 4:**
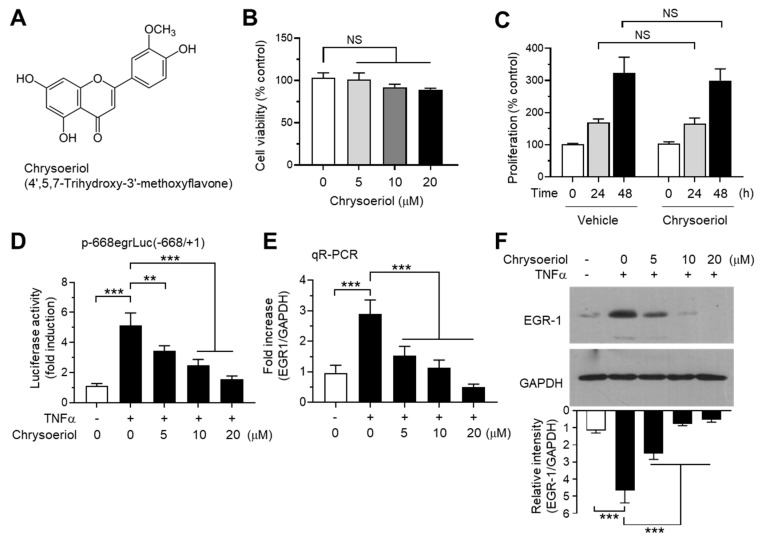
Effect of chrysoeriol on EGR-1 expression. (**A**) Chemical structure of chrysoeriol. (**B**) Cell viability assay. MCF-7 cells were treated with vehicle (DMSO) or increasing concentrations of chrysoeriol (0–20 μM) for 24 h, and cell viability was determined using the Cell Counting Kit-8 (CCK-8). Graph bars represent the mean ± SD (*n* = 3). NS, not significant by Sidak’s multiple comparisons test. (**C**) MCF-7 cells were treated with vehicle (DMSO) or 20 μM chrysoeriol for different periods (0, 24, and 48 h), and cell proliferation was measured using an ELISA Colorimetric kit for the detection of 5-bromo-2′-deoxyuridine (BrdU) incorporation. Graph bars represent the mean ± SD (*n* = 3). NS, not significant by Sidak’s multiple comparisons test. (**D**) MCF-7 cells were transfected with 0.1 µg of *EGR1* promoter–reporter p-668egrLuc(–668/ + 1). After 48 h, the cells were treated with 10 ng/mL TNFα in the presence or absence of chrysoeriol (0, 5, 10, 20 μM) for an additional 8 h, and the luciferase reporter activities were measured. (**E**,**F**) MCF-7 cells were pretreated with chrysoeriol (0, 5, 10, and 20 μM) for 30 min, followed by treatment with 10 ng/mL TNFα. After 1 h, cells were harvested and the *EGR1* mRNA levels or protein levels were measured by qR-PCR (**E**) or immunoblot analysis (**F**), respectively. Band intensities were measured using the ImageJ software. Bars represent the mean ± SD (*n* = 3). ** *p* < 0.01, *** *p* < 0.001 by Dunnett’s multiple comparisons test. qR-PCR, quantitative real-time PCR.

It has been reported that some flavons and flavanones differentially regulate CYP19 expression; hesperetin stimulates, while luteolin inhibits CYP19 aromatase expression [40]. To further identify novel bioactive compounds that inhibit CYP19 expression, we collected 39 commonly occurring natural flavonoids, including 26 flavones and 13 flavanones (Table S1), and tested their effects on TNFα-induced EGR1 promoter activity. MCF-7 cells were transfected with the EGR1 promoter-reporter plasmid p-668egrLuc(–668/+ 1), and the cells were pretreated with the compounds for 30 min before TNFα stimulation. After 18 h, the cells were harvested, and EGR1 promoter–reporter activity was measured. We observed that 3,7-dimethoxyflavone, chrysoeriol, geraldol, kaempferide, tricetin, galangin, quercetagetin, myricetin, naringin, and hesperidin inhibited TNFα-induced EGR1 promoter activity by more than 70%, whereas tectochrysin, 7,8-dimethoxyflavone, negletin, tricetin trimethylether, dimethylpinocembrin, and eriodictyol enhanced EGR1 promoter activity by more than 70% (Table S1). Among them, chrysoeriol (4′,5,7-trihydroxy-3′-methoxyflavone; CAS no. 491-71-4; Figure 4A) exhibited the strongest inhibitory activity (97.0 ± 2.20%). Chrysoeriol is present in lemon, Rooibos tea, pepper, and green vegetables such as celery, and exerts antioxidant, anti-inflammatory, and antitumor effects [41,42,43,44,45]. It has been reported that chrysoeriol inhibits CYP1B1 enzyme activity responsible for hydroxylation of 17β-estradiol in MCF-7 breast cancer cells [46]. However, it is unknown whether chrysoeriol modulates CYP19 expression. Therefore, we selected chrysoeriol and further characterized its mode of action with respect to inhibition of EGR-1-mediated CYP19 expression.

### 2.5. Chrysoeriol Inhibited TNFα-Induced EGR-1 Expression at the Transcript Level

Chrysoeriol did not affect cell viability (Figure 4B) and cell proliferation (Figure 4B) at concentrations below 20 μM in MCF-7 cells. At these concentrations, we performed a dose-dependent experiment to verify the effect of chrysoeriol on the inhibition of EGR1 promoter activity. Chrysoeriol significantly inhibited TNFα-induced EGR1 promoter activity in a dose-dependent manner (Figure 4D). To further evaluate the inhibitory effect of chrysoeriol on the EGR-1 expression, we measured EGR1 mRNA levels using qR-PCR. CYP19 mRNA levels increased by 2.90 ± 0.458-fold in response to TNFα stimulation, which was significantly reduced by 1.53 ± 0.306-, 1.13 ± 0.252-, and 0.500 ± 0.100-fold in the presence of 5, 10, and 20 μM chrysoeriol, respectively (Figure 4E). Moreover, chrysoeriol significantly prevented the TNFα-induced accumulation of EGR-1 protein in a dose-dependent manner (Figure 4F). At 10 μM, chrysoeriol inhibited 83.3% of TNFα-induced EGR-1 accumulation (from 4.66 ± 0.741-fold to 0.780 ± 0.106-fold). Therefore, the inhibitory effect of chrysoeriol on TNFα-induced EGR1 mRNA and protein expression follows a similar pattern, indicating that chrysoeriol prevents TNFα-induced EGR-1 expression at the transcript level in MCF-7 breast cancer cells.

### 2.6. Chrysoeriol Inhibited TNFα-Induced CYP19 Expression at the Transcript Level

**Figure 5:**
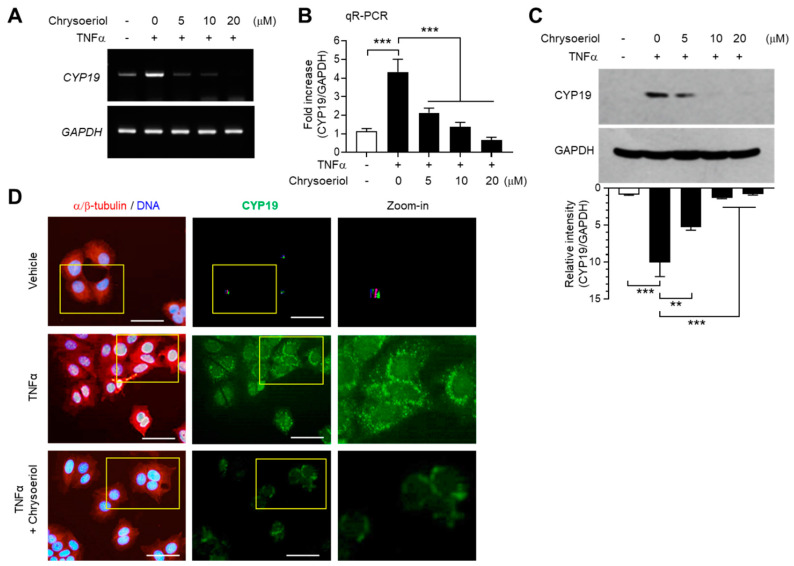
Effect of chrysoeriol on CYP19 expression. (**A**–**C**) MCF-7 cells were pretreated with chrysoeriol (0–20 μM) for 30 min, followed by treatment with 10 ng/mL TNFα. After 24 h, CYP19 expression was determined by RT-PCR (**A**), qR-PCR (**B**), and immunoblot analysis (**C**). GAPDH was used as an internal control. Band intensities were measured using the ImageJ software. Bars represent the mean ± SD (*n* = 3). ** *p* < 0.01, *** *p* < 0.001 by Dunnett’s multiple comparisons test. qR-PCR, quantitative real-time PCR. (**D**) MCF-7 cells cultured on coverslips were treated with 10 ng/mL TNFα in the absence or presence of 10 μM chrysoeriol, followed by fixation, permeabilization, and incubation with primary antibodies specific to α/β-tubulin and CYP19 aromatase for 2 h. After washing, Alexa Fluor 555 (for α/β-tubulin; red signal) and Alexa Fluor 488 (for CYP19; green signal) secondary antibodies were used to probe the samples for 30 min. Nuclear DNAs were stained with Hoechst 33258 for 10 min (blue signal). Fluorescently labeled cells were viewed under an EVOS FL fluorescence microscope. Nuclear DNA and α/β-tubulin were overlaid (left panels). Yellow boxed regions are zoomed-in on the far right. Scale bars represent 20 μm.

We next determined whether inhibition of EGR-1 expression by chrysoeriol is functionally linked to the suppression of CYP19 expression. TNFα-induced CYP19 mRNA expression was reduced by chrysoeriol, as revealed by RT-PCR (Figure 5A) and qR-PCR (Figure 5B) analyses. Ten micromoles of chrysoeriol reduced TNFα-induced CYP19 mRNA expression by 64% (from 4.33 ± 0.681-fold to 1.37 ± 0.252-fold). Densitometric analysis of immunoblots revealed that chrysoeriol inhibited TNFα-induced CYP19 aromatase accumulation by approximately 86% (from 10.1 ± 1.95-fold to 1.39 ± 0.0808-fold) (Figure 5C). Immunofluorescence revealed that cells stained with anti-CYP19 aromatase antibody were abundant after TNFα stimulation; however, their numbers reduced substantially in the presence of chrysoeriol (Figure 5D). These results suggest that chrysoeriol reduces TNFα-induced accumulation of CYP19 aromatase expression via suppression of EGR-1 transcriptional ability in MCF-7 cells.

### 2.7. ERK1/2 MAPK Pathway Mediated TNFα-Induced EGR-1 Expression in MCF-7 Cells

**Figure 6:**
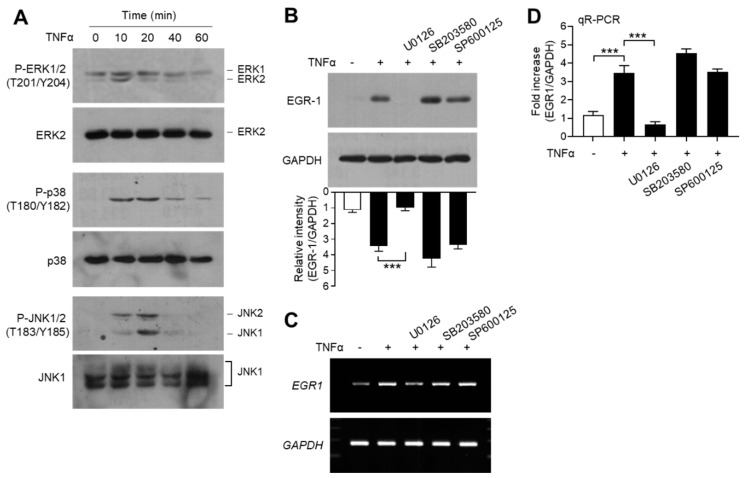
Effect of mitogen-activated protein kinase (MAPK) inhibition on EGR-1 expression. (**A**) Serum-starved MCF-7 cells (cultured in medium containing 0.5% serum for 24 h) were treated with 10 ng/mL TNFα for various durations (0–60 min). Total protein extracts were analyzed by immunoblotting using antibodies against phospho- extracellular signal-regulated kinase 1/2 (ERK1/2) (Thr202/Tyr204), phospho- c-Jun N-terminal kinase 1/2 (JNK1/2) (Thr183/Tyr185), and phospho-p38 kinase (Thr180/Tyr182). Corresponding total MAPK protein was used as an internal control. (**B**–**D**) Serum-starved MCF-7 cells were pretreated with 5 μM U0126, 10 μM SP600125, or 10 μM SB203580 for 30 min, followed by treatment with 10 ng/mL TNFα. After 60 min, cells were harvested and EGR-1 expression was examined by immunoblotting (**B**), RT-PCR (**C**), and qR-PCR (**D**). GAPDH was used as an internal control. Band intensities were measured using the ImageJ software. Bars represent the mean ± SD (*n* = 3). *** *p* < 0.001 by Dunnett’s multiple comparisons test. qR-PCR, quantitative real-time PCR.

EGR-1 expression is regulated by mitogen-activated protein kinase (MAPK) pathways, including extracellular signal-regulated kinase 1/2 (ERK1/2; MAPK3/MAPK1), c-Jun N-terminal kinase 1/2 (JNK1/2; MAPK8/9), and p38 kinase (MAPK14), in MCF7 breast cancer cells [47]. We confirmed the effect of MAPK pathways on TNFα-induced EGR-1 expression in MCF-7 cells. Serum-starved MCF-7 cells were treated with TNFα, and MAPK activation was determined using phospho-specific antibodies. We observed that TNFα stimulated the phosphorylation of all three MAPKs—ERK1/2 on Thr-201/Tyr-204, JNK1/2 on Thr-183/Tyr-185, and p38 kinase on Thr-180/Tyr-182—within 10 min, compared to the unstimulated basal levels (Figure 6A). Chemical inhibitors were used to assess the potential involvement of MAPK signaling in TNFα-induced EGR-1 expression. Pretreatment of MCF-7 cells with the MAPK/ERK kinase 1/2 (MEK1/2; MAP2K2) inhibitor U0126 reduced the levels of TNFα-induced EGR-1 proteins, whereas the p38 kinase inhibitor SB203580 and the JNK inhibitor SP600125 did not reduce the accumulation of TNFα-induced EGR-1 protein (Figure 6B). Likewise, RT-PCR (Figure 6C) and qR-PCR (Figure 6D) yielded results that were comparable to those from immunoblot analysis. Therefore, ERK1/2 MAPK may play a critical role in TNFα-induced EGR-1 expression in MCF-7 cells.

### 2.8. Chrysoeriol Inhibited the ERK1/2 MAPK Pathway to Block TNFα-Induced CYP19 Expression in MCF-7 Cells

**Figure 7:**
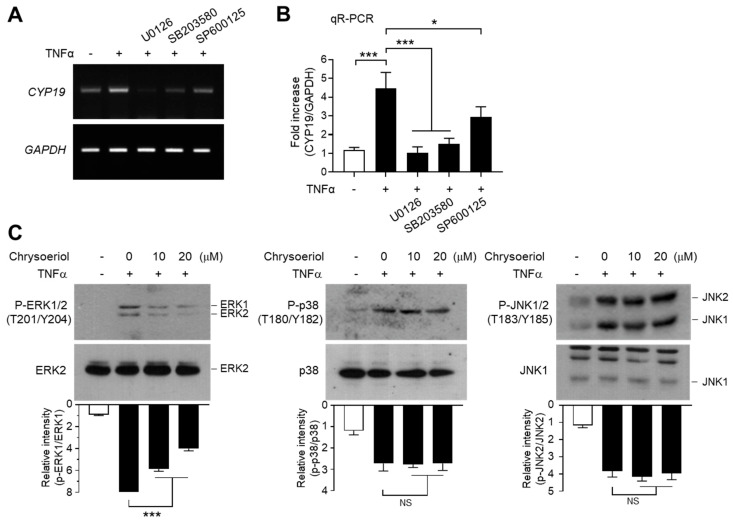
Effect of chrysoeriol on the inhibition of MAPK and CYP19 expression. (**A**,**B**) Serum-starved MCF-7 cells were pretreated with 5 μM U0126, 10 μM SP600125, or 10 μM SB203580 for 30 min, followed by treatment with 10 ng/mL TNFα. After 24 h, cells were harvested and *CYP19* mRNA expression was examined by RT-PCR (**A**) and qR-PCR (**B**). GAPDH was used as an internal control. (**C**) Serum-starved MCF-7 cells were pretreated with chrysoeriol (0, 10, and 20 μM) for 30 min, followed by treatment with 10 ng/mL TNFα. After 10 min (phospho-ERK1/2) or 20 min (phospho-JNK1/2 and phospho-p38), cells were harvested, and immunoblotting was performed using antibodies against phospho-ERK1/2 (Thr202/Tyr204), phospho-JNK1/2 (Thr183/Tyr185), and phospho-p38 kinase (Thr180/Tyr182). Band intensities were measured using the ImageJ software, and phospho-proteins were normalized to corresponding total proteins. Bars represent the mean ± SD (*n* = 3). NS, not significant; * *p* < 0.05, *** *p* < 0.001 by Dunnett’s multiple comparisons test. qR-PCR, quantitative real-time PCR.

We next determined the effect of MAPK inhibition on TNFα-induced CYP19 mRNA expression. Pretreatment with U0126 inhibited the ability of TNFα to induce CYP19 mRNA expression, as revealed by RT-PCR (Figure 7A) and qR-PCR (Figure 7B). Notably, SB203580 and SP600125 significantly inhibited TNFα-induced CYP19 mRNA expression as well. These data suggest that all three MAPK pathways are involved in TNFα-induced CYP19 mRNA expression. It seems likely that ERK1/2 mediates EGR-1-dependent CYP19 expression, whereas JNK1/2 and p38 kinase induce CYP19 expression independent of EGR-1.

It has been demonstrated that prostaglandin E2 stimulates all three MAPKs (ERK, JNK, and p38 kinase); however, JNK and p38 kinase, though not ERK, are necessary for CYP19 aromatase expression in adipocyte fibroblasts [48]. In contrast, follicle-stimulating hormone-induced CYP19 aromatase expression is mediated by the phosphatidylinositol 3-kinase signaling pathway, whereas the ERK MAPK pathway inhibits estrogen production in testis Sertoli cells [49]. As CYP19 transcription is controlled in a tissue-specific manner [50], it seems likely that ERK, JNK, and p38 MAPKs might contribute to tissue-specific regulation of CYP19 transcription.

Finally, we sought to address the involvement of the MAPK pathways in chrysoeriol-induced suppression of CYP19 expression. Pretreatment with chrysoeriol significantly abrogated TNFα-induced ERK1/2 phosphorylation in a dose-dependent manner, while the same was not applicable to JNK1/2 and p38 kinase (Figure 7C). These data suggest that chrysoeriol inhibits TNFα-induced CYP19 expression via selective inhibition of ERK1/2-mediated EGR-1 expression.

## 3. Materials and Methods 

### 3.1. Materials

Natural flavonoid compounds were procured from Indofine Chemical Co. Inc. (Hillsborough, NJ, USA). Human recombinant TNFα, MAP kinase inhibitors (U0126, SB203580, and SP600125), and Hoechst33258 were obtained from Sigma-Aldrich (Saint Louis, MO, USA). The Luciferase Assay System was procured from Promega (Madison, WI, USA). Antibodies against GAPDH (cat. no. sc-32233; dilution 1:1000), JNK1 (cat. no. sc-571; dilution 1:1000), ERK2 (cat. no. sc-154; dilution 1:1000), and EGR-1 (dilution 1:1000; cat. no. sc-189) were purchased from Santa Cruz Biotechnology (Santa Cruz, CA, USA). Anti-phospho-ERK1/2 (Thr202/Tyr204, cat. no. #9101; dilution 1:500), -phospho-p38 (Thr180/Tyr182, cat. no. #9211; dilution 1:500), -p38 (cat. no. 9212; dilution 1:1000), and -phospho-JNK1/2 (Thr183/Tyr185, cat. no. #9251; dilution 1:500) antibodies were from Cell Signaling Technology (Danvers, MA, USA). Anti-CYP19 aromatase antibody (cat. no. MCA2077S; dilution 1:500) was purchased from Bio-Rad (Hercules, CA, USA), and anti-α/β tubulin antibody (cat. no. MA1-19400; dilution 1:200) was from ThermoFisher Scientific (Waltham, MA, USA). In terms of secondary antibodies, horse radish-conjugated anti-rabbit (cat. no. #7074; dilution 1:3000) and -mouse (cat. no. #7076; dilution 1:3000) antibodies were from Cell Signaling Technology, and Alexa Fluor 488- conjugated anti-rabbit (cat. no. A-11094; dilution 1:300) and Alexa Fluor 555-conjugated anti-mouse (cat. no. A-21422; dilution 1:300) antibodies were obtained from Invitrogen (Carlsbad, CA, USA).

### 3.2. Cell Culture

Human MCF-7 breast cancer cells were procured from the American Type Culture Collection (Manassas, VA, USA). Cells were maintained in Dulbecco’s modified Eagle’s medium/Nutrient Mixture F-12 (ThermoFisher Scientific, Waltham, MA, USA) supplemented with 10% fetal bovine serum (Hyclone, Logan, UT, USA) and penicillin–streptomycin (Sigma-Aldrich). MCF-7 cells expressing scrambled control shRNA (shCT) or EGR-1 shRNA (shEgr1) were used as described in a previous report [51].

### 3.3. Reverse Transcription PCR (RT-PCR)

MCF-7 cells were treated with TNFα or chrysoeriol, and total RNA was extracted using a TRIzol RNA extraction kit (Invitrogen, Carlsbad, CA, USA). One microgram of RNA was reverse-transcribed into complementary DNA (cDNA) using an iScript cDNA synthesis kit (Bio-Rad, Hercules, CA, USA). RT-PCR was performed using the reverse transcriptase enzyme according to the manufacturer’s instructions (Promega). The gene-specific PCR primers used were as follows:

- *EGR1* forward primer, 5′-CAG CAG TCC CAT TTA CTC AG-3′;
- *EGR1* reverse primer, 5′-GAC TGG TAG CTG GTA TTG-3′;
- *CYP19* forward primer, 5′-CAC ACC AGA GAA CCA GGC TAC AAG-3′;
- *CYP19* reverse primer, 5′-TGA ATG TTG CTT TTC CAC CTC C-3′;
- glyceraldehyde-3-phosphate dehydrogenase (*GAPDH*) forward primer, 5′-CCA AGG AGT AAG AAA CCC TGG AC-3′;
- *GAPDH* reverse primer, 5′-GGG CCG AGT TGG GAT AGG G-3′.

PCR conditions were as follows: hold for 5 min at 94 °C, followed by 30 cycles consisting of denaturation at 94 °C (30 s), annealing at 55 °C (30 s), and elongation at 72 °C (1 min). The amplified products were electrophoresed on a 2% agarose gel using ethidium bromide and were detected under UV light.

### 3.4. Quantitative Real-Time PCR (qR-PCR)

Quantitation of mRNA levels was conducted using the quantitative real-time PCR (qR-PCR) approach using an iCycler iQ system with an iQ SYBR Green Supermix kit (Bio-Rad, Hercules, CA, USA) according to the manufacturer’s recommendations. Validated commercial qR-PCR primers and SYBR Green-based fluorescent probes specific for CYP19 mRNA (id: qHsaCIP0026454) and GAPDH mRNA (id: qHsaCEP0041396) were obtained from Bio-Rad. PCR conditions were as follows: denaturation at 95 °C for 2 min, followed by 40 cycles using a step program (95 °C for 10 s and 60 °C for 45 s). The relative expression levels of CYP19 mRNA were normalized to those of GAPDH using the software provided by the manufacturer.

### 3.5. Immunoblotting

Cells were lysed in a buffer consisting of 20 mM Hydroxyethyl piperazine Ethane Sulfonicacid (HEPES, pH 7.2), 1% Triton X-100, 10% glycerol, 150 mM NaCl, 10 μg/mL leupeptin, and 1 mM phenylmethylsulfonyl fluoride. The protein extracts were separated by 10% SDS-PAGE and transferred to nitrocellulose membranes (Bio-Rad). The blots were incubated with the appropriate primary and secondary antibodies and developed using an Amersham ECL Western Blotting Detection Kit (GE Healthcare Life Science, Chicago, IL, USA).

### 3.6. EGR1 Promoter Reporter Assay

The MCF-7 cells seeded onto 12-well plates were transfected with 0.1 µg of the EGR1 promoter construct p-668egrLuc(–668/+ 1) [52] using Lipofectamine 2000 reagent (Invitrogen) according to the manufacturer’s instructions. To monitor the transfection efficiency, we included a pRL^®^-null plasmid (50 ng) encoding *Renilla* luciferase in all transfections. At 48 h post-transfection, the levels of firefly luciferase and *Renilla* luciferase activities were measured sequentially from the same sample using the Dual-Glo Luciferase Assay System (Promega), as described previously [38]. The relative luciferase activity in the untreated cells was designated 1. The luminescent signal was detected and measured using a dual luminometer Centro LB960 (Berthold Tech, Bad Wildbad, Germany). For the screening of flavonoid compounds inhibiting TNFα-induced EGR1 promoter activity, we calculated the inhibitory activity by the following formula:

(1) $$\mathrm{Inhibitory}\;\mathrm{activity}\;(\%)\;=\;\left[1-\frac{P_{F}-p_{b}}{P_{T}-P_{b}}\right]\times 100$$

where *P_F_* = flavonoid-induced EGR1 promoter activity, *Pb* = unstimulated basal EGR1 promoter activity, and *P_T_* = TNFα-induced EGR1 promoter activity.

### 3.7. Immunofluorescence

MCF-7 cells cultured on coverslips were either treated with phosphate-buffered saline (PBS) or 10 ng/mL TNFα in the presence or absence of chrysoeriol for 24 h, followed by fixation, permeabilization, and incubation with primary antibodies. Samples were probed with primary antibodies against α/β-tubulin and CYP19 aromatase for 2 h, followed by probing with Alexa Fluor 555- (for α/β-tubulin; red signal) and Alexa Fluor 488-conjugated (for CYP19; green signal) secondary antibodies for an additional 30 min. Nuclear DNAs were stained with Hoechst 33258 for 10 min (blue signal). Fluorescent cells were examined under an EVOS FL fluorescence microscope (Advanced Microscopy Group; Bothell, WA, USA).

### 3.8. Statistical Analysis

The data are plotted as means with SD. Statistical comparisons were performed using a one-way ANOVA followed by Dunnett’s or Sidak’s multiple comparisons test with the GraphPad Prism V8.3.1 software (GraphPad Software, San Diego, CA, USA). A *p*-value *<* 0.05 was considered statistically significant.

## 4. Conclusions

CYP19 aromatase overexpression is associated with malignant phenotypes in the human breast [1]. However, aberrant expression of CYP19 aromatase is not regulated by the proliferation of tumor cells or clinical course; instead, it occurs as a result of interaction between tumor cells and stromal cells [53,54]. Accordingly, we observed that untreated MCF-7 cells expressed low levels of CYP19 aromatase, while TNFα stimulation resulted in an increase in CYP19 mRNA levels. Additionally, we observed that pretreatment with chrysoeriol prior to TNFα treatment substantially prevented TNFα-induced CYP19 expression via the downregulation of ERK MAPK-mediated EGR-1 expression. TNFα is the major inflammatory cytokine produced by adipose tissues, tumor-associated fibroblasts, and various inflammatory cells infiltrating the tumor tissues [22]. It is well established that localized TNFα in the tumor microenvironment promotes inflammation-associated tumors in most solid tumors, including breast cancer [55,56]. Additionally, estrogen induces EGR-1 expression in MCF-7 cells [57]. This gives rise to the possibility that EGR-1-dependent signaling loop and the TNFα-EGR-1–CYP19–estrogen-EGR-1 axis could result in persistent activation of estrogen production, thereby facilitating the development of ER-positive breast cancer in the tumor microenvironment. Besides CYP19 aromatase, EGR-1 enhances paclitaxel-induced multi-drug resistance by upregulation of P-glycoprotein, an ATP-dependent efflux pump [58], and mediates TNFα-induced GROα and MMP-9 expression [38,39]. Furthermore, inhibition of EGR-1 by DNAzyme inhibits fibroblast growth factor-dependent angiogenesis in breast cancer [37]. Therefore, suppression of EGR-1 and CYP19 aromatase expression by dietary chrysoeriol in the breast tumor microenvironment may be beneficial as a preventive agent or as a chemotherapeutic adjuvant against estrogen receptor-positive breast cancer.

Chrysoeriol is present in several foods and vegetables [41,42,43,44,45]. Many dietary phenolics are generally poorly bioavailable, limiting their distribution in systemic tissues of their native form, mainly as glycosides and complex oligomeric structures [59]. In general, methylation protects the flavonoids from widespread conjugation, and methylated flavonoids have improved intestinal absorption and metabolic stability compared to unmethylated forms [60]. Chrysoeriol contains a methoxy group attached to the C3’ atom of the flavonoid backbone. The pharmacokinetics profile of circulating chrysoeriol is not fully characterized yet. However, the oral bioavailability and tissue distribution of chrysoeriol could be indirectly predicted through the study of luteolin (3′,4′,5,7-tetrahydroxyflavone). Luteolin is a common catechol-type flavonoid present in many types of edible plants and medicinal herbs [61], possessing a wide range of biological effects, including anti-inflammatory and anti-cancer activities [62]. However, luteolin is hardly detected and is mainly present in the metabolites of glucuronides and methylated forms in vivo [63]. Glucuronidation by uridine 5′-diphospho-glucuronosyltransferases (UGTs) and methylation by catechol-O-methyltransferases (COMTs) are two main metabolic pathways of luteolin in animals and humans [64,65]. Chrysoeriol is generated by the methoxylation of luteolin at the C3’ atom (luteolin-3′-methoxy ether) by catechol-O-methyltransferase (COMT) [66]. Chrysoeriol has been detected in rat plasma samples after oral administration of luteolin [63] and is broadly distributed to the lungs, kidney, spleen, muscle, and heart, but found to be undetectable in the brain, within 1 h after oral administration [67], suggesting that circulating chrysoeriol can reach various target tissues. The anti-breast cancer efficacy of chrysoeriol in vivo is closely related to its concentration in breast cancer tissues. The distribution of chrysoeriol in the breast tissue has not been well characterized. As the distribution of phenolic metabolites in breast tissue is similar to that observed in plasma [59] and the fact that chrysoeriol exerts chemopreventive cancer effects in mouse mammary organ culture [68], we suggest that circulating chrysoeriol could reach primary breast cancer tissues and exert anti-cancer effects. To provide evidence-based potential of chrysoeriol as a functional food or nutrition supplement in the prevention or treatment of breast cancer, further physiologically relevant in vivo studies, including bioavailability, metabolism, and tissue distribution of dietary chrysoeriol and its derived metabolites, are necessary [59].

In conclusion, the present study demonstrates that chrysoeriol reduces TNFα-induced upregulation of CYP19 aromatase expression via inhibition of ERK MAPK-mediated EGR-1 expression in MCF-7 breast cancer cells. Chrysoeriol might serve as a beneficial supplement or adjuvant for the prevention or treatment of estrogen receptor-positive breast cancer with the potential to inhibit local estrogen production in the tumor microenvironment in breast cancer.

## Supplementary Materials

The following are available online at https://www.mdpi.com/1422-0067/21/20/7523/s1, Table S1: Inhibitory effects of flavonoids on TNFα-induced EGR1 promoter activity.

## Abbreviations

CYP19	Cytochrome P450 19 gene
EGR-1	Early growth respone-1
ER	Estrogen receptor
ERK	Extracellular signal-regulated kinase
GAPDH	Glyceraldehyde 3-phosphate dehydrogenase
JNK	c-Jun N-terminal kinase
MAPK	Mitogen-activated protein kinase
qR-PCR	Quantitative real-time polymerase chain reaction
RT-PCR	Reverse-transcription polymerase chain reaction
TNFα	Tumor necrosis factor alpha
