# Supplementary material for: Chrysoeriol Prevents TNFα-Induced CYP19 Gene Expression via EGR-1 Downregulation in MCF7 Breast Cancer Cells
Source: Int J Mol Sci. 2020 Oct 12;21(20):7523. doi: 10.3390/ijms21207523 (PMC7588959; doi:10.3390/ijms21207523)
Supplement: Supplementary file 1 [file ijms-21-07523-s001.pdf]

Table S1. Inhibitory effects of flavonoids on TNFα-induced EGR1 promoter activity

	CAS No*	Common Name	Structure Name	IA**
1.	525-82-6	Flavone	Flavone	0.5±0.1
2.	491-78-1	Primuletin	5-Hydroxyflavone	6.1±0.5
3.			3,7-Dimethoxyflavone	94.0±4.3
4.	520-28-5	Tectochrysin	5-Hydroxy-7-Methoxyflavone	-115.3±11.0
b5.	65548-54-1		7,8-Dimethoxyflavone	-98.0±7.3
6.	29550-13-8	Negletein	5,6-Dihydroxy-7-methoxyflavone	-356.7±111.5
7.	437-64-9	Genkwanin	5,4'-Dihydroxy-7-methoxyflavone	3.8±1.2
8.	520-36-5	Apigenin	5,7,4'-Trihydroxyflavone	64.7±7.6
9.	491-67-8	Baicalein	5,6,7-Trihydroxyflavone	-27.3±6.6
10.	528-48-3	Fisetin	3,7,3',4'-Tetrahydroxyflavone	41.3±6.6
11.	491-70-3	Luteolin	5,7,3',4'-Tetrahydroxyflavone	35.3±7.4
12.	491-71-4	Chrysoeriol	4',5,6-Trihydroxy-3'-methoxyflavone	97.0±2.2
13.	529-53-3	Scutellarein	5,6,7,4'-Tetrahydroxyflavone	56.7±5.4
14.	21511-25-1	Geraldol	3,7,4'-Trihydroxy-3'-methoxyflavone	79.7±7.9
15.	491-54-3	Kaempferide	3,5,7-Trihydroxy-4'-methoxyflavone	79.3±6.9
16.	7555-80-8		3,5,7,2',4'-Pentamethoxyflavone	-4.0±0.8
17.	2306-27-6	Sinensetin	5,6,7,3',4'-Pentamethoxyflavone	38.0±6.7
18.	520-31-0	Tricetin	5,7,3',4',5'-Pentahydroxyflavone	74.3±9.7
19.	53350-26-8	Tricetin pentamethyl ether	5,7,3',4',5'-Pentamethoxyflavone	-28.0±5.0
20.	18103-42-9	Tricetin trimethylether	5,7-Dihydroxy-3',4',5'-trymethoxyflavone	-81.7±8.5
21.	2196-14-7		7,4'-Dihydroxyflavone	-61.0±9.8
22.	548-83-4	Galangin	3,5,7-Trihydroxyflavone	86.3±11.9
23.	90-18-6	Quercetagetin	3,4,6,7,3',4'-Hexahydroxyflavone	80.7±8.6
24.	489-35-0	Gossypetin	3,5,7,8,3',4'-Hexahydroxyflavone	24.3±7.8
25.	529-44-2	Myricetin	3,5,7,3',4',5'-Hexahydroxyflavone	89.3±4.6
26.	29043-07-0		5,6,7,3',4',5'-Hexamethoxyflavone	55.0±10.2
27.	480-41-1	Naringenin	5,7,4'-Trihydroxyflavanone	1.4±0.2
28.	10236-47-2	Naringin	4′,5,7-Trihydroxyflavanone 7-rhamnoglucoside	70.7±10.6
29.	520-33-2	Hesperetin	5,7,3'-Trihydroxy-4'-methoxyflavanone	-32.3±9.2
30.	68745-38-0	Pinocembrin	5,7-Dihydroxyflavanone	43.7±8.3
31.	1036-72-2	Dimethylpinocembrin	5,7-Dimethoxyflavanone	-108.7±5.8
32.	552-58-9	Eriodictyol	5,7,3',4'-Tetrahydroxyflavanone	-76.7±5.4
33.	13241-32-2	Neoeriocitrin	Eriodictyol-7-O-neohesperidoside	55.0±7.3
34.	6515-36-2		7-Hydroxyflavanone	-28.7±3.4
35.	21785-09-1		7-Methoxyflavanone	14.3±2.9
36.	520-26-3	Hesperidin	3′,5,7-Trihydroxy 4′-methoxyflavanone 7-rutinoside	81.7±7.7
37.	480-37-5	Pinostrobin	5-Hydroxy-7-methoxyflavanone	45.0±7.8
38.	36052-37-6	(S)-Alpinetin	(S)-7-Hydroxy-5-methoxyflavanone	-7.3±1.2
39.	2957-21-3	Sakuranetin	5,4'-Dihydroxy-7-methoxyflavanone	43.0±4.9

* CAS No; Chemical Abstract Service Register Number. ** IA: Inhibitory activity (%) of flavonoids against TNFα-induced *EGR1* promoter activation. Data value, mean±S.D. (n = 3); A negative value indicates an increase in TNFα-induced *EGR1* promoter activity; A positive value indicates a decrease in TNFα-induced *EGR1* promoter activity.
